# Surgical treatment of gastrointestinal stromal tumors

**DOI:** 10.25122/jml-2025-0168

**Published:** 2026-01

**Authors:** Andreea Maria Godja, Adrian Mihai Eftimie, Cristian Balalau, Irina Balescu, Nicolae Bacalbasa

**Affiliations:** 1 Department of Visceral Surgery, Center of Digestive Diseases and Liver Transplantation, Fundeni Clinical Institute, Bucharest, Romania; 2 Department of Visceral Surgery, Carol Davila University of Medicine and Pharmacy, Bucharest, Romania; 3 Department of Visceral Surgery, St. Pantelimon Emergency Clinical Hospital, Bucharest, Romania; 4 Department of Visceral Surgery, Center of Excellence in Translational Medicine, Fundeni Clinical Institute, Bucharest, Romania

**Keywords:** gastric GIST, gastric gastrointestinal stromal tumors, laparoscopic, endoscopic, GIST, gastrointestinal stromal tumors, SDH, succinate dehydrogenase, NF1, neurofibromatosis type 1, CT, computed tomography, EUS, endoscopic ultrasound, PET, positron emission tomography, FNA, fine-needle aspiration, EFTR, endoscopic full-thickness resection, STER, submucosal tunneling endoscopic resection, ESTD, endoscopic submucosal tunnel dissection, LECRS, laparoscopic and endoscopic cooperative resection of submucosal tumors, ESD, endoscopic submucosal dissection, LECS, laparoscopic and endoscopic cooperative surgery, NEWS, non-exposed endoscopic wall-inversion surgery, PFS, progression-free survival, RFS, recurrence-free survival, DSS, disease-specific survival, OS, overall survival, NIH, National Institutes of Health, AFIP, Armed Forces Institute of Pathology, TKI, tyrosine kinase inhibitors

## Abstract

Gastrointestinal stromal tumors (GISTs) are the most common mesenchymal tumors of the gastrointestinal tract, though they represent a small proportion of overall gastrointestinal malignancies. Originating from the interstitial cells of Cajal, GISTs exhibit a broad spectrum of clinical behavior, ranging from indolent lesions to aggressive malignancies that can arise throughout the gastrointestinal tract, most frequently in the stomach and small intestine, and rarely in extra-gastrointestinal sites such as the omentum, mesentery, or retroperitoneum. Surgical management, particularly for gastric GISTs, increasingly favors minimally invasive approaches, including laparoscopic, robotic, and endoscopic techniques, while preserving oncologic safety. Complete surgical excision with negative margins remains the cornerstone of curative therapy for primary gastric GISTs. Indications for surgery are most often related to symptomatic gastric GISTs at initial presentation. Optimal patient care relies on a multidisciplinary strategy integrating surgical management, pathology, imaging, risk stratification, and targeted medical therapies, ensuring individualized treatment plans and improved outcomes.

## Introduction

Gastrointestinal stromal tumors (GISTs) constitute the predominant mesenchymal neoplasm of the gastrointestinal tract, representing roughly 80% of these tumors and about 0.1%–3% of all gastrointestinal malignancies [1,2]. Approximately one-third demonstrate malignant behavior [3]. While GISTs can develop anywhere along the gastrointestinal tract, the stomach is the most frequent site (about 50–60%), followed by the small intestine (20%–30%), the colon and rectum (5%–10%), and rarely the esophagus (<5%) [4,5]. First recognized as a distinct clinicopathological entity in the 1980s, GISTs were initially misclassified as smooth muscle tumors. However, advances in immunohistochemistry and the identification of activating *KIT* and platelet-derived growth factor receptor alpha (*PDGFRA*) mutations have since established their unique identity as a separate tumor type [6,7]. Gastric GISTs are often discovered incidentally during upper endoscopy or imaging performed for unrelated reasons. They usually protrude into the gastric lumen, though they can also present as endophytic or exophytic lesions. Symptomatic cases most commonly involve bleeding when the lesion has surface ulceration [4,7,8].

### Epidemiology and molecular pathogenesis

GISTs are the most common mesenchymal tumors of the gastrointestinal tract, with an estimated annual incidence of 10–15 cases per million people [9,10]. The majority of GISTs harbor mutations in the *KIT* gene, specifically in exon 11, while approximately 5–10% have mutations in the *PDGFRA* gene [11,12]. Mutations in the PDGFRA exon 18 (D842V) are associated with resistance to Imatinib, a standard treatment for GISTs. Additionally, tumors with succinate dehydrogenase (SDH) deficiency and those associated with neurofibromatosis type 1 (NF1) exhibit distinct clinicopathological features and may require tailored therapeutic approaches [9,13,14].

### Diagnosis, staging, and indications for surgery of gastric GISTs

The evaluation of suspected gastric GISTs requires a comprehensive, multimodal approach [15]. Contrast-enhanced computed tomography (CT) remains the cornerstone for assessing tumor size, anatomical location, and potential metastatic dissemination. Upper gastrointestinal endoscopy provides direct mucosal visualization, whereas endoscopic ultrasound (EUS) enables precise characterization of the lesion layer of origin, echotexture, and internal heterogeneity. Positron emission tomography (PET) may be selectively applied for lesions with high metabolic activity or in the context of neoadjuvant therapy planning. Histopathologic confirmation is primarily indicated when neoadjuvant therapy is under consideration (Table 1) [10,16-18].

**Table 1:** Diagnostic modalities in gastric GISTs

Modality	Role	Strengths	Limitations
CT (contrast-enhanced)	Baseline imaging, staging	Widely available	Limited in small lesions
EUS	Lesion size, wall origin, biopsy	High diagnostic accuracy	Operator-dependent
PET	Assess response, detect metastasis	High sensitivity	Cost, not routine
Biopsy (EUS-FNA/TTNB)	Required for TKI planning	Confirms diagnosis	May not predict malignant potential

### Surgical management of gastric GISTs

**Figure 1:**
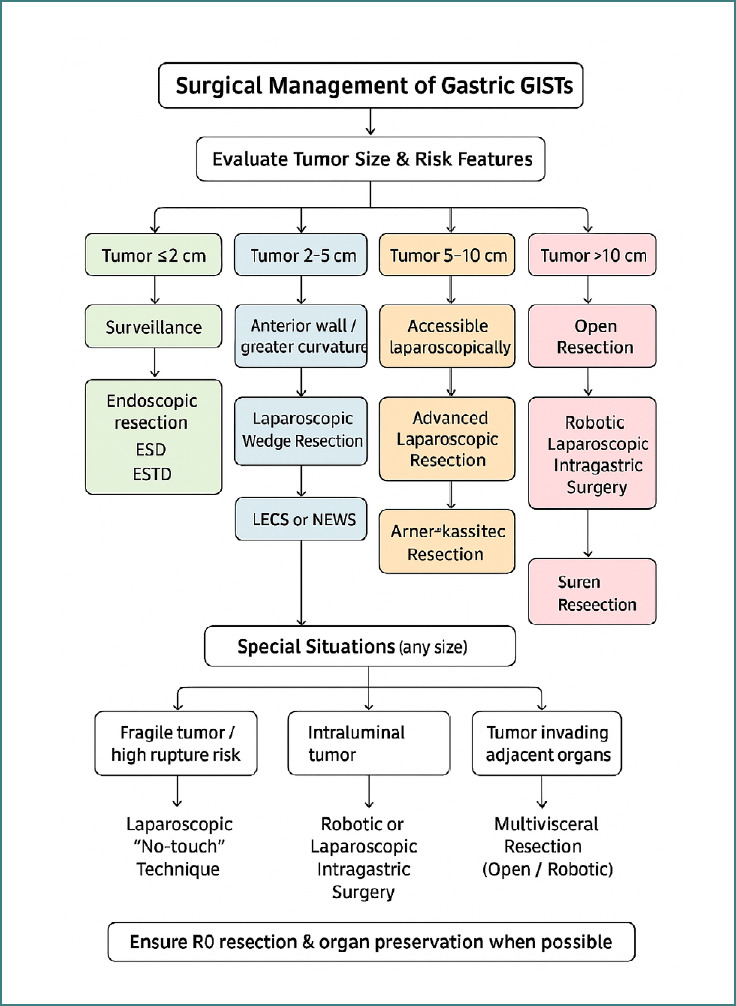
Surgical management of GISTs: size, location, and technique selection. ESD, Endoscopic Submucosal Dissection; ESTD, Endoscopic Submucosal Tunnel Dissection; LECS, Laparoscopic and Endoscopic Cooperative Surgery; NEWS, Non-Exposed Endoscopic Wall-Inversion Surgery.

Surgical resection remains the primary treatment modality for localized gastric GISTs. The goal is complete gross resection with negative margins, as this is associated with improved prognosis [19]. Minimally invasive techniques, including laparoscopic and robotic-assisted surgeries, have become increasingly prevalent due to their advantages in reducing postoperative pain, shortening hospital stays, and enhancing recovery times. However, the choice between open and minimally invasive approaches depends on tumor size, location, and surgeon expertise (Figure 1) [20-23].

Historically, the surgical management of gastric GISTs was dominated by open techniques. Wedge or segmental resections via laparotomy were standard, providing direct visualization and careful handling of the tumor, particularly in cases of large, highly vascularized, or friable lesions, thereby minimizing the risk of intraoperative rupture [24-26]. For large endophytic tumors, gastrotomy-based excision was performed, involving opening the gastric wall, excising the tumor with a margin of normal mucosa, and meticulous closure of the gastrotomy. While effective, these procedures were associated with prolonged recovery, increased postoperative pain, and a higher risk of wound-related complications [27-29]. Enucleation or 'shell-out' techniques, historically considered for small, well-circumscribed tumors, are now contraindicated due to the potential for pseudocapsule disruption, gastric wall perforation, and peritoneal dissemination [30,31].

#### Contemporary surgical strategies have increasingly favored minimally invasive, organ-preserving approaches, including

- **Laparoscopic wedge and segmental resections** – suitable for exophytic or easily accessible tumors, providing rapid recovery and minimal postoperative discomfort.
- **Endoscopic full-thickness resection (EFTR)** – indicated for tumors with significant intraluminal growth while preserving gastric wall integrity.
- **Submucosal tunneling endoscopic resection (STER)** – particularly useful for submucosal tumors near the gastroesophageal junction, allowing intraluminal excision with a protective mucosal tunnel.
- **Endoscopic submucosal tunnel dissection (ESTD)** – enables en bloc resection of larger submucosal lesions while maintaining the overlying mucosa.
- **Laparoscopic and endoscopic cooperative resection of submucosal tumors (LECRS)** – combines laparoscopy and endoscopy for precise excision in anatomically challenging regions.
- **Hybrid laparoscopic-endoscopic approaches** – employed for complex tumors requiring both intraluminal and external manipulation to minimize trauma.
- **Robotic-assisted resection** – provides enhanced dexterity and visualization for tumors in difficult-to-access locations, particularly near the gastroesophageal junction or pylorus.

In selected cases, particularly when tumors are adjacent to the pylorus or gastroesophageal junction, intraoperative stenosis may necessitate additional interventions such as gastrojejunostomy or vagotomy to ensure gastric emptying and preserve function [32-34]. The evolution from open, radical resections to minimally invasive, organ-sparing techniques reflects both technological advancements and an improved understanding of GIST biology [35,36]. Modern approaches prioritize avoiding pseudocapsule disruption, preserving gastric function, and maintaining oncologic safety, while retaining the capacity to manage complex or high-risk tumors when required [37-40].

### Gastric GISTs trial studies

Recent clinical trials have expanded the understanding of both surgical and pharmacological management of gastric GISTs. While numerous pharmacotherapy-focused studies are ongoing, particularly investigating novel KIT inhibitors and combination regimens for advanced or resistant GISTs, surgical intervention trials remain limited. Collectively, these trials aim to refine surgical strategies, optimize patient outcomes, and integrate emerging pharmacological therapies, shaping the future landscape of gastric GIST management (Table 2).

**Table 2:** Recent and ongoing clinical trials in advanced gastric GISTs

Study	Phase	Therapy	Target population	Key findings
**INTRIGUE [41]**	Phase III	Ripretinib vs. Sunitinib	Advanced GIST post-imatinib	Ripretinib showed superior progression-free survival (PFS) in patients with KIT exon 11 + 17/18 mutations.
**StrateGIST 1 [42]**	Phase 1/1b	IDRX-42 (pan-KIT inhibitor)	Metastatic GIST resistant to prior TKIs	Promising anti-tumor activity across various KIT mutations; ongoing dose-expansion phase.
**THE-630 Study [43]**	Phase 1	THE-630 (novel agent)	Advanced GIST	Evaluating safety, efficacy, and pharmacokinetics; details forthcoming.
**Ripretinib vs. Sunitinib [44]**	Phase III	Ripretinib vs. Sunitinib	Advanced GIST post-imatinib	Comparing efficacy in patients with specific KIT mutations; study ongoing.
**177Lu-NeoB [45]**	Phase I/IIa	177Lu-NeoB (radiolabeled peptide)	GIST with GRPR overexpression	Assessing safety and anti-tumor activity; ongoing recruitment.
**Avelumab + Axitinib (AXAGIST) [46]**	Early Phase	Avelumab + Axitinib	Pretreated metastatic GIST	3-month PFS rate of 57.1%; further studies needed.
**Binimetinib + Imatinib [47]**	Phase II	Binimetinib + Imatinib	Advanced GIST	10-year follow-up shows sustained clinical benefit in PFS and overall survival (OS).

GIST, gastrointestinal stromal tumor; TKI, tyrosine kinase inhibitor; PFS, progression-free survival; OS, overall survival; GRPR, gastrin-releasing peptide receptor The above information is available on the www.clinicaltrials.gov website.

### Follow-up and prognosis of resected gastric GISTs

Recent studies have provided valuable insights into the follow-up strategies and long-term prognosis of patients who have undergone resection for gastric GISTs. Zhang *et al*. conducted a large-scale retrospective study involving 532 patients with very low-risk and low-risk GISTs who underwent endoscopic resection. The study reported five-year recurrence-free survival (RFS) rates of 98.5% and 95.9%, respectively, and five-year disease-specific survival (DSS) rates of 100% in both groups. These findings imply that routine postoperative surveillance, incorporating imaging and endoscopic procedures, may not be required for these low-risk patients [48].

In contrast, a multicentre cohort study led by the Italian Sarcoma Group involving 737 patients with low-risk GISTs who underwent surgical resection revealed a relapse rate of 5.7% over a median follow-up period of 69.2 months. Notably, recurrences were observed even after more than 10 years, indicating that long-term monitoring remains important. However, the benefit of routine follow-up in low-risk cases is yet to be determined [49].

Moreover, a study by Liu *et al*. analysed long-term survival outcomes in 1,223 patients with GISTs measuring 5-10 cm, comparing endoscopic and surgical treatments. The results showed that the two treatment methods had similar 5-year (86.5% vs. 83.5%) and 10-year (70.4% vs. 66.7%) overall survival rates, suggesting that endoscopic treatment can be an effective option for tumors in this size range [50].

Collectively, these studies underscore the importance of individualized follow-up strategies based on tumor risk classification and treatment modality (Table 3). While low-risk gastric GISTs may not require intensive postoperative surveillance, high-risk cases necessitate more vigilant monitoring to detect potential recurrences. Ongoing research is needed to establish standardized follow-up protocols and to determine the optimal duration and frequency of monitoring for patients with resected gastric GISTs.

**Table 3:** Follow-up and prognosis of resected gastric GISTs: recent evidence from clinical studies

Study	Year	Population & Intervention	Key findings	Follow-up/Prognosis
Zhang *et al*. [48]	2024	532 patients with very low-risk and low-risk gastric GISTs; endoscopic resection	5-year RFS: 98.5% (very low-risk), 95.9% (low-risk); 5-year DSS: 100%	Suggested that routine postoperative surveillance may not be necessary for low-risk patients
Italian Sarcoma Group [49]	2023	737 low-risk GIST patients; multicenter cohort study	5-year DFS: 95.5%; 10-year DFS: 93.4%; GIST-specific survival: 98.1%; OS: 91.0%; relapse in 5.7%	Highlights the importance of long-term monitoring, median follow-up 69.2 months
Liu *et al*. [50]	2024	Gastric GISTs 5–10 cm; comparison of endoscopic vs surgical treatment	5-year OS: 86.5% (endoscopic) vs 83.5% (surgical); 10-year OS: 70.4% vs 66.7%	Endoscopic treatment can be a viable alternative for tumors within this size range

GIST, gastrointestinal stromal tumor; RFS, recurrence-free survival; DSS, disease-specific survival; DFS, disease-free survival; OS, overall survival

### Risk stratification and prognostic models

Accurate risk stratification is essential for predicting recurrence and guiding postoperative management. Traditional models, such as the National Institutes of Health (NIH) consensus criteria and the Armed Forces Institute of Pathology (AFIP) criteria, consider factors like tumor size, mitotic index, and location (Table 4) [51,52]. Recent studies have integrated molecular data into these models, recognizing the prognostic significance of specific mutations. For instance, tumors with *KIT* exon 11 mutations generally have a worse prognosis, while those with *PDGFRA* D842V mutations may have an indolent course but are resistant to imatinib therapy [53-55].

**Table 4:** Risk stratification models for gastric GISTs

Model	Parameters Considered	Strengths	Limitations
NIH consensus [51]	Tumor size, mitotic index, and location	Widely used, simple to apply	Does not incorporate molecular data
AFIP criteria [52]	Tumor size, mitotic index, and location	Provides detailed risk assessment	May overestimate risk in some cases
Molecular-based [53-55]	KIT/PDGFRA mutations, SDH status	Incorporates genetic information	Requires molecular testing

### Multimodal therapy

The advent of tyrosine kinase inhibitors (TKIs), particularly Imatinib, has revolutionized the treatment of GISTs. Adjuvant therapy with Imatinib has been shown to improve recurrence-free survival in high-risk patients [13,21,56]. The optimal duration of adjuvant therapy remains a subject of ongoing research, with studies suggesting benefits from extended treatment durations [53]. Neoadjuvant Imatinib therapy is utilized to reduce tumor size and facilitate surgical resection, especially in cases where complete resection would otherwise be challenging [12,57]. Resistance to Imatinib, particularly in tumors with *PDGFRA* D842V mutations, necessitates alternative therapeutic strategies, including second-line TKIs like Sunitinib and Regorafenib [57].

Recent advancements in the surgical management of gastric GISTs have led to improved patient outcomes. Minimally invasive surgical techniques offer benefits in terms of recovery and cosmetic outcomes, though they require specialized training and equipment. The integration of molecular data into risk-stratification models enables more personalized treatment approaches, potentially improving prognostic accuracy. However, challenges remain in determining the optimal duration of adjuvant therapy and in managing resistance to standard TKIs. Future research should focus on prospective studies to validate molecular-based risk models and to evaluate the efficacy of novel therapeutic agents in the management of gastric GISTs [7].

## Conclusion

Surgical resection remains the cornerstone of treatment for gastric GISTs. Advancements in minimally invasive surgical techniques and the incorporation of molecular data into risk stratification have enhanced the management of these tumors. Ongoing research into the optimal use of adjuvant and neoadjuvant therapies, as well as the development of strategies to overcome resistance to TKIs, will further improve patient outcomes. A multidisciplinary approach, involving surgeons, medical oncologists, and pathologists, is essential for the effective management of gastric GISTs.
